# Genomewide Analyses Define Different Modes of Transcriptional Regulation by Peroxisome Proliferator-Activated Receptor-β/δ (PPARβ/δ)

**DOI:** 10.1371/journal.pone.0016344

**Published:** 2011-01-19

**Authors:** Till Adhikary, Kerstin Kaddatz, Florian Finkernagel, Anne Schönbauer, Wolfgang Meissner, Maren Scharfe, Michael Jarek, Helmut Blöcker, Sabine Müller-Brüsselbach, Rolf Müller

**Affiliations:** 1 Institute of Molecular Biology and Tumor Research (IMT), Philipps University, Marburg, Germany; 2 Helmholtz Centre for Infection Research (HZI), Braunschweig, Germany; Institute of Genetics and Molecular and Cellular Biology, France

## Abstract

Peroxisome proliferator-activated receptors (PPARs) are nuclear receptors with essential functions in lipid, glucose and energy homeostasis, cell differentiation, inflammation and metabolic disorders, and represent important drug targets. PPARs heterodimerize with retinoid X receptors (RXRs) and can form transcriptional activator or repressor complexes at specific DNA elements (PPREs). It is believed that the decision between repression and activation is generally governed by a ligand-mediated switch. We have performed genomewide analyses of agonist-treated and PPARβ/δ-depleted human myofibroblasts to test this hypothesis and to identify global principles of PPARβ/δ-mediated gene regulation. Chromatin immunoprecipitation sequencing (ChIP-Seq) of PPARβ/δ, H3K4me3 and RNA polymerase II enrichment sites combined with transcriptional profiling enabled the definition of 112 *bona fide* PPARβ/δ target genes showing either of three distinct types of transcriptional response: (I) ligand-independent repression by PPARβ/δ; (II) ligand-induced activation and/or derepression by PPARβ/δ; and (III) ligand-independent activation by PPARβ/δ. These data identify PPRE-mediated repression as a major mechanism of transcriptional regulation by PPARβ/δ, but, unexpectedly, also show that only a subset of repressed genes are activated by a ligand-mediated switch. Our results also suggest that the type of transcriptional response by a given target gene is connected to the structure of its associated PPRE(s) and the biological function of its encoded protein. These observations have important implications for understanding the regulatory PPAR network and PPARβ/δ ligand-based drugs.

## Introduction

Peroxisome proliferator-activated receptors (PPARs) are nuclear receptors with essential functions in lipid, glucose and energy metabolism, cell differentiation as well as inflammatory and metabolic disorders [1]–[4]. The PPARα, PPARβ/δ and PPARγ subtypes activate their target genes through binding to specific DNA elements (PPREs) as obligatory heterodimers with the retinoid X receptor (RXR). Their transcriptional activity is modulated by certain lipids, fatty acid derivatives and subtype-selective synthetic ligands that have been developed as potential drugs for the treatment of human metabolic diseases [5]. PPRE-bound PPAR complexes have two distinct functions, i.e., transcriptional repression and transcriptional activation. Agonistic ligands induce a conformational change in PPARs that favors the association with coactivators and the dissociation of corepressors [6]. Several PPAR-associated corepressors have been identified [7]–[12], but their precise function remains largely obscure. Likewise, it is unclear whether all genes targeted by a given PPAR subtype are regulated in a similar way, or whether distinct regulatory mechanisms govern the expression of different sets of PPAR target genes.

A genomewide binding site analysis of PPARγ during adipocyte differentiation by chromatin immunoprecipitation sequencing (ChIP-Seq) revealed an exchange of PPARβ/δ for PPARγ, presumably switching from repressive to activating complexes on the promoters of key target genes [13]. Bioinformatic analyses of ChIP-chip data also revealed the interaction of C/EBP factors with DNA elements in the vicinity of PPARγ binding sites in adipocytes [14], while in macrophages an interplay of PPARγ with both C/EBP and the Ets family member PU.1 was observed [15]. A recent ChIP-chip study of PPARα binding sites in HepG2 hepatoma cells provides evidence for a crosstalk between PPRE-bound PPARα and SREBP signaling at some target gene promoters [16]. The same study also points to an interaction between PPARα and STAT transcription factors in PPARα-mediated transcriptional repression, consistent with previous observation made with individual target genes. In a different context, PPRE-associated PPARβ/δ has been described to interact with, and mediate the SUMOylation of KLF5, leading to NCoR/SMRT dissociation, CBP recruitment and consequently transcriptional activation [17].

It has previously been shown that PPARs regulate the differentiation, function and proliferation of myofibroblasts in different model systems [18], [19]. These include tumor-bearing *Ppard* null mice, which show a hyperplastic tumor stroma associated with a strongly increased differentiation towards myofibroblasts [20]. A role for PPARβ/δ in myofibroblasts is further suggested by an extensive crosstalk with transforming growth factor-β (TGFβ) signaling, which affects the composition of chromatin complexes at common target genes [21], [22]. In the present study, we used human myofibroblast-like cells as a model system for a genome-wide analysis of PPARβ/δ-regulated transcription. By combining ChIP-Seq analysis with genome-wide transcriptional profiling we show that, contrary to the prevailing opinion, transcriptional repression and activation are not merely determined by the availability of agonistic ligands, but are governed by gene-specific mechanisms. Based on these data we define different modes of transcriptional regulation by PPRE-bound PPARβ/δ, and correlate these with the structure of PPARβ/δ sites and the biological function of the encoded proteins.

## Results and Discussion

### Genomewide identification of PPARβ/δ enrichment sites in WPMY-1 cell chromatin

**Figure 1:**
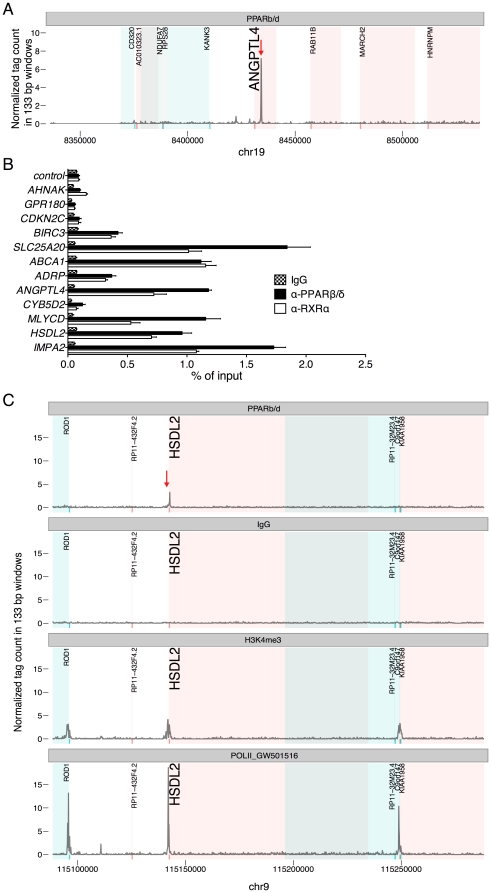
Genomewide identification of PPARβ/δ binding sites in WPMY-1 cells by ChIP-Seq. (**A**) PPARβ/δ enrichment at the genomic *ANGPTL4* locus determined by ChIP-Seq. (**B**) ChIP-qPCR analysis of PPARβ/δ and RXRα binding at 12 genomic loci identified by ChIP-Seq. (**C**) PPARβ/δ, H3K4me3 and RNA polymerase II enrichment peaks detected by ChIP-Seq at the *HSDL2* locus.

Standard quantitative ChIP-qPCR was initially used to analyze chromatin from WPMY-1 cells for PPARβ/δ occupancy of the well-characterized PPAR-responsive enhancer of the *ANGPTL4* gene, which harbors a cluster of 3 functional PPREs in the third intron at +3.5 kb relative to the transcriptional start site (TSS) [21], [23]. We found an ∼20-fold enrichment of PPARβ/δ at the PPRE-containing intronic enhancer and a low background signal within 20 kb flanking the TSS (Kaddatz *et al*, 2010). Deep sequencing of this DNA was then performed using an Illumina genome analyzer II. A total of 20,777,020 reads mappable to unique locations on the human genome were obtained. Analysis of the ChIP-Seq data set using the MACS peak calling algorithm (Zhang *et al*, 2008) identified a total of 4,542 enrichment peaks (Dataset S1). This is exemplified by the profile for the *ANGPTL4* locus in Figure 1A, and for the *SLC25A20* and *CDKN2* loci in Figures S1 and S2. The *ANGPTL4* ChIP-Seq data are in perfect agreement with the pattern observed by ChIP-qPCR (Kaddatz *et al*, 2010). Further validation was obtained by ChIP-qPCR for a sample of 40 peaks, which also demonstrated the presence of RXRα at the same genomic loci (Figure 1B and data not shown).

In order to assess the potential biological relevance of the PPARβ/δ enrichment sites, we analyzed the 4,542 enrichment peaks for associations with genomic loci (single nucleotide polymorphisms; SNPs) linked to phenotypes via genome wide association studies (GWAS) (Ramagopalan, 2010). Within this dataset reflecting 47 diseases and other common traits there was a significant enrichment in three groups (*p*< = 0.05; Benjamini-Hochberg corrected), i.e., cholesterol, HDL cholesterol and bipolar disorder (Dataset S2). This is in excellent agreement with the known physiological role function of PPARβ/δ in lipid metabolism [1], [4] and its reported linkage with bipolar disorder [24].

Most PPARβ/δ enrichment sites were found inside or maximum 25 kb upstream of transcribed genomic regions (*n* = 3,544; 78%; Ensembl 58). These sites were located close to transcriptional start sites (−5000 bp upstream or within the first exon/intron of a TSS (*n* = 2,220; 49% of all peaks), within intragenic regions (*n* = 868; 19%) or in non-transcribed upstream sequences of the TSS (*n* = 456; 10%). The remaining 22% (*n* = 998) were assigned to more distant regions (>25 kb relative to the nearest TSS).

**Figure 2:**
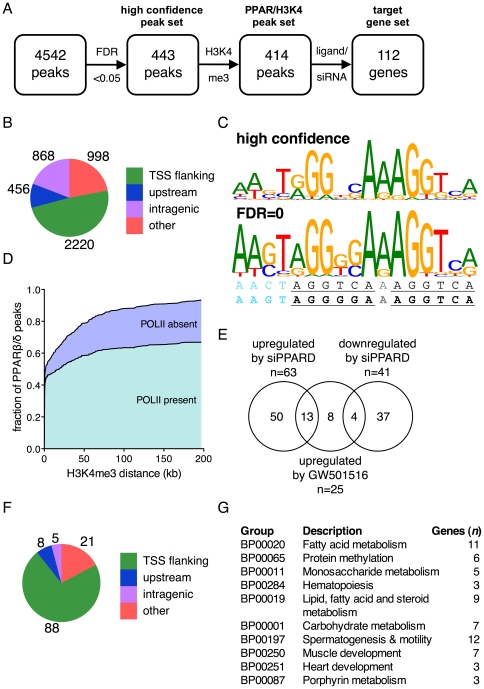
Genomewide identification of PPARβ/δ target genes by combining ChIP-Seq and transcriptional profiling. (**A**) Flow chart showing consecutive steps of bioinformatic analysis for the definition of high confidence PPARβ/δ-regulated genes (target gene set). These genes are characterized by one or more peaks with an FDR<0.05 that is/are located within 200 kb of a database-defined gene (Ensembl release 58), a cluster of H3K4me3 marks, RNA polymerase II enrichment and transcriptional responsiveness to *PPARD* siRNA and/or the agonist GW501516. (**B**) Distribution of genomewide PPARβ/δ binding for all 4,542 peaks identified by ChIP-Seq. *TSS flanking* is defined as regions from −5000 bp to the 3′ end of the first intron, *upstream* regions are located within −25 kb of a transcriptional start site (TSS). (**C**) Consensus sequence identified by *de novo* motif search (MEME) [25] of ChIP-Seq in high confidence (top) and FDR = 0 peaks (bottom). The line beneath shows the published consensus sequence [27]; the bottom line (boldface) shows the refined sequence derived from the present study. (**D**) Overlap between high confidence PPARβ/δ peaks, H3K4me3 marks and RNA polymerase II enrichment detected by parallel ChIP-Seq experiments. (**E**) Venn diagram showing the overlap between genes regulated by *PPARD* siRNA or activated by GW501516. (**F**) Analysis of PPARβ/δ peak distribution for the target gene set. (**G**) Panther biological process (BP) classification of the target gene set. The 10 most enriched BP terms describing biological processes affected by PPARβ/δ are shown.

All peaks with an FDR = 0 were used for a *de novo* motif search (MEME) [25], which yielded a 17-bp consensus sequence (Figure 2C; bottom image and bottom line). This motif is composed of a typical direct repeat 1 (DR1) flanked by a 4-bp extension at the PPAR-binding 5′-half site [26], which is consistent with, and refines, the previously proposed consensus sequence (Figure 2C; upper line) [27]. We then searched all 443 enrichment peaks with an FDR<0.05 (‘high confidence peak set’; Dataset S3; Figures 2A) for a 17-bp consensus sequence using MEME, which yielded a similar motif matching 287 (65%) of the 443 sequences (Figure 2C; top image).

### Correlation of PPARβ/δ enrichment sites with promoter regions

The conventional, simple approach of associating peaks with the nearest gene defined in a database often yields uncertain results, because frequently multiple genes are found in the vicinity of an enrichment peak, and transcriptional start sites in databases can be incorrectly assigned. To circumvent this problem we took a different approach. In parallel to PPARβ/δ ChIP-Seq, we performed global analyses of histone H3 lysine-4 trimethylation (H3K4me3) and RNA polymerase II enrichment (Datasets S4 and S5) as markers for active or inducible proximal promoters [28], [29]. These ChIP-Seq data were aligned with the data set of high confidence PPARβ/δ enrichment peaks, as shown for the *HSDL2* locus in Figure 1C and two other loci in Figures S1 and S2. This correlation led to the delineation of 414 peaks with H3K4me3 clusters within 200 kb of the PPARβ/δ binding sies (PPAR/H3K4 peak set; Figure 2A), corresponding to 93.5% of all high confidence peaks (Figure 2D). The majority of these genomic regions also showed the presence of RNA polymerase II (70%; Figures 1C, 2D, S1 and S3). Those PPARβ/δ ChIP-Seq peaks that did not co-localize with H3K4me3 clusters may be associated with enhancers [28], located in genes switched off in myofibroblasts, or result from non-functional interaction sites [30].

### Identification of PPARβ/δ target genes by combining ChIP-Seq and transcriptional profiling

For genomewide expression profiling of *PPARD*-depleted WPMY-1 cells, we used a pool of validated, *PPAR* subtype-specific siRNAs, which in a previous study was shown to inhibit *PPARD* expression in WPMY-1 myofibroblastic cells by >80% and to interfere with the recruitment of PPARβ/δ to the *ANGPTL4* PPREs *in vivo* [21]. This siRNA pool also inhibited the transcriptional activation of a PPRE-luciferase reporter construct by the PPARβ/δ agonist GW5101516, which was rescued by PPARβ/δ overexpression, arguing against potential off-target effects (Figure S3). Microarray analysis of WPMY-1 cells exposed to control or *PPARD* siRNA and, in parallel, in the presence or absence of GW501516 (Datasets S6 and S7) enabled the delineation of a subgroup of 118 expression-correlated peaks in the PPAR/H3K4 set, corresponding to 112 genes (Figure 2A, Dataset S8). These genes (“target gene set”) showed a ≥1.5-fold change in expression after *PPARD* knockdown or a ≥1.2-fold change after application of GW501516, and were therefore considered *bona fide* PPARβ/δ target genes (Figure 2E). Surprisingly, only a fraction of genes (*n = *13; 12%) were both induced by *PPARD* siRNA and activated by GW501516, suggesting that an agonist-induced switch between repression and activation is not the rule.

We next determined the location of PPARβ/δ enrichment sites relative to the linked gene within the target gene set. As expected, there was a slight increase in sites near or within transcribed genes (Figure 2F; 83% TSS-flanking, intragenic or upstream within 25 kb) compared to the unfiltered peak set (Figure 2B; 78%). Panther Biological Pathway term analysis [31] via the DAVID knowledge database [32] showed that the majority of the 10 most enriched terms describing biological processes affected by PPARβ/δ were associated with lipid and carbohydrate metabolism (Figure 2G; Dataset S9). This expected result further supports the target gene set defined in the present study. The same analysis also identified several other groups of target genes that are of potential interest in view of PPARβ/δ's non-metabolic functions, including hematopoiesis and muscle/heart development.

### Different modes of target gene regulation

**Figure 3:**
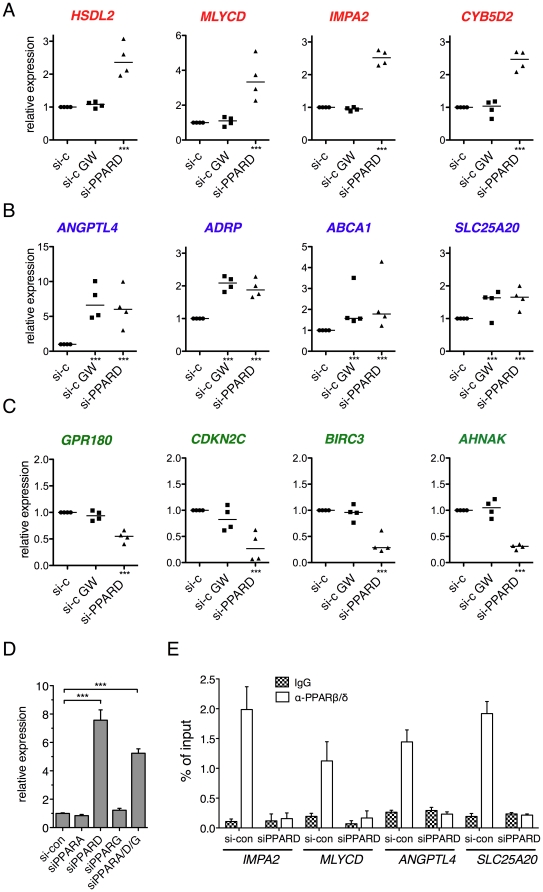
Identification of different types of transcriptional responses to PPARβ/δ depletion and ligands. (**A–C**) Differential responses to GW501516 and *PPARD* siRNA of PPARβ/δ target genes, classified as type I (**A**), type II (**B**) and type III (**C**) responses. WPMY-1 cells were treated as indicated and analyzed as in Figure 1. Data from four biological replicates are shown. Individual data points represent the average of 3 technical replicates. Horizontal lines indicate the median of 4 biological replicates. (**D**) PPAR subtype-specific repression of the *ANGPTL4* gene by PPARβ/δ. WPMY-1 cells were transfected with *PPARA, PPARG* or *PPARD* siRNA pools, or a combination of all three pools (triple knock-down), and relative *ANGPTL4* mRNA levels were measured by RT-qPCR. The efficiencies and subtype specificities of the siRNA pools are shown in Figure S4. (**E**) Effect of *PPARD* siRNA treatment on PPARβ/δ recruitment to the *IMPA2, MLYCD, ANGPTL4, SLC25A20, BIRC3* and *GPR180* genes.

**Figure 4:**
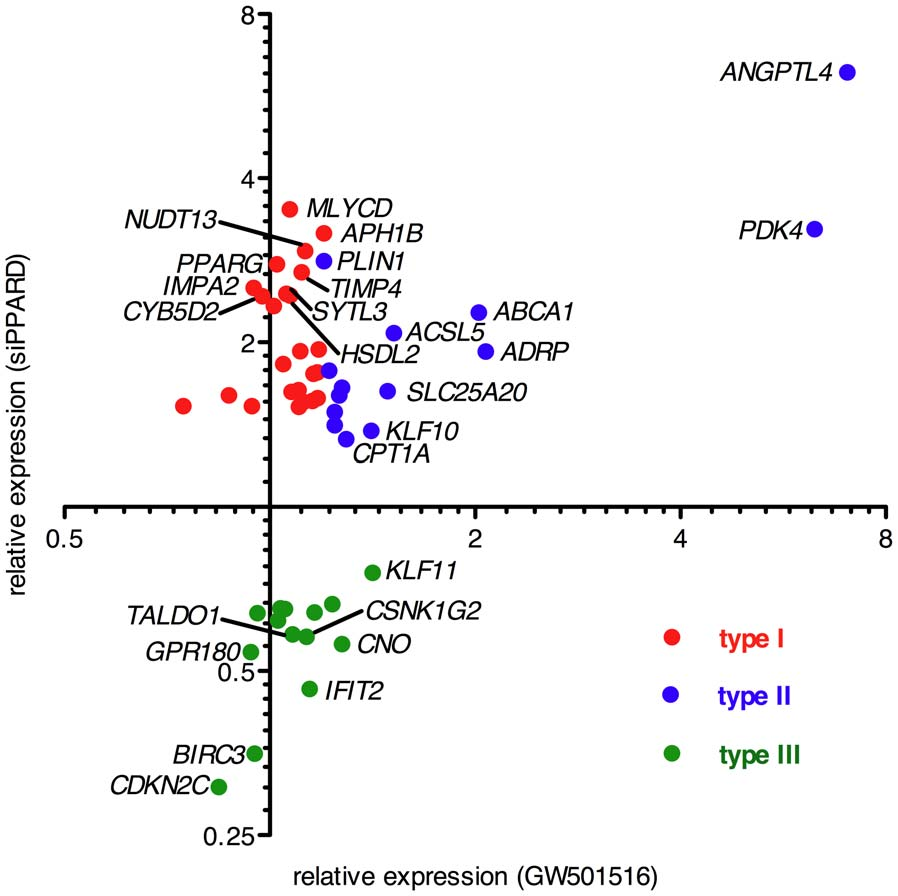
Correlation of *PPARD* siRNA and GW501516 mediated gene regulation for the validated gene set. Red: type I response, upregulated (≥1.5-fold) by *PPARD* siRNA and unaffected by GW501516; blue: type II response, upregulated by *PPARD* siRNA and induced by GW501516 (≥1.2-fold); green: type III response, down-regulated (≥1.5-fold) by *PPARD* siRNA. Each data point represents the average of 4 biological replicates as in Figure 3A–C.

The response of PPARβ/δ target genes to GW501516 (relative to solvent) and *PPARD* siRNA (relative to control siRNA) was verified by RT-qPCR for a total of 53 genes (Dataset S10), as illustrated by the examples in Figure 3A–C. Based on these data, we defined three different transcriptional responses (Figure 4): upregulation (≥1.5-fold) by *PPARD* siRNA, but no induction (<1.2-fold) by ligand (type I; *n = *25; red data points); upregulation by *PPARD* siRNA and induction by ligand (type II; *n = *14; blue data points); down-regulation by *PPARD* siRNA, and either no response or weak induction by GW501516 (type III; *n = *14; green data points). This categorization was also reproducible with a different PPARβ/δ agonist (L165,041), and ligand induction was abolished by *PPARD* siRNA (data not shown).

The data also indicate that the magnitude of induction by ligand approaches the effect of PPARβ/δ depletion for individual genes showing a type II response (Figures 3A–C, 4). Since the concentration of GW501516 used (0.3 µM) causes the maximally achievable transcriptional induction (our unpublished observation), this suggests that ligand-induced transcription results to a large extent from the release of a PPARβ/δ-RXR repressor complex. This postulated PPAR subtype-specific repressive function is consistent with the observation that the siRNA-mediated depletion of PPARα or PPARγ did not have any detectable effect on *ANGPTL4* expression (Figure 3D). Furthermore, a triple knockdown of all three PPAR subtypes had a similar effect as the selective PPARβ/δ knockdown (Fig. 3D), suggesting that PPARα and PPARγ do not activate target genes that are normally repressed by PPARβ/δ. This finding is important in view of the fact that PPARβ/δ depletion leads to a compensatory upregulation of PPARα and PPARγ (Figure S4).

To obtain further evidence for a direct role of PPARβ/δ in both ligand-induced and ligand-independent responses, we analyzed the effect of *PPARD* siRNA on the chromatin association of PPARβ/δ at representative target genes, i.e., *IMPA2* and *MLYCD* (type I) and *ANGPTL4* and *SLC25A20* (type II). The data in Fig. 3E shows that in each case siRNA treatment led to clear loss of PPARβ/δ irrespective of the different transcriptional outcomes (Fig. 3A, B). These results support the conclusion that ligand-independent responses are directly linked to the recruitment of PPARβ/δ to the respective target genes.

The ligand-independent activation of type III response genes by PPARβ/δ may suggest a role for endogenous ligands produced by WPMY-1 cells. This is, however, unlikely in view of the fact that type II response genes are activated by GW501516 under the identical experimental conditions (Fig. 3B, C). Furthermore, a typical type III response was also seen in HepG2 hepatoma cells with several tested genes, including *BIRC3* and *AHNAK* (our unpublished observation). Finally, a clear transcriptional induction of *ANGPTL4* was also seen with agonists that have a substantially lower affinity than the synthetic GW501516, such as 15-HETE [33], arguing against the possibility that WPMY-1 cells produce particularly high levels of endogenous PPARβ/δ ligands.

### Structural features associated different types of response

**Figure 5:**
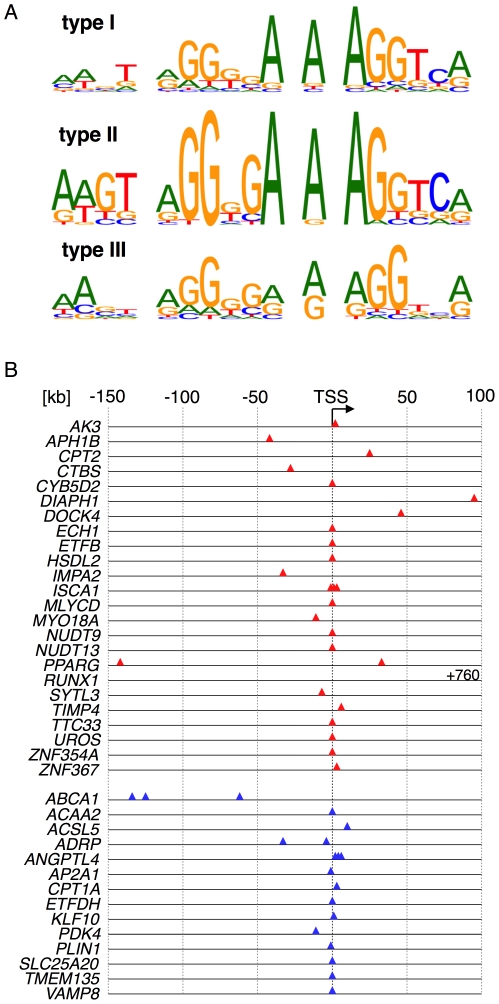
Structural features of PPARβ/δ target genes showing type I or type II responses. (**A**) Consensus PPRE motifs in ChIP-Seq peak areas of validated type I, II and III response genes derived by best-fit alignment of peak sequences with the FDR = 0 motif in Fig. 2C. (**B**) Locations of PPREs in PPARβ/δ enrichment peaks (type I: red; type II: blue; numbers relative to the TSS). All sites downstream of the TSS are intragenic.

This validated gene set was used for the identification of potential response-selective DNA sequence motifs within the associated ChIP-Seq peaks. *De novo* motif search in peak areas associated with both type I and type II responses identified motifs (Figure 5A) that closely resembled the consensus DR1 sequence defined above (see Figure 2C). However, only the type II-associated motif perfectly matched both the DR-1 motif and the 5′ extension. In contrast, no correlations were found between the type of response of a given gene, the position of PPARβ/δ enrichment peak(s) and the number of PPREs (Figure 5B). It is therefore tempting to speculate that the structurally different PPREs may contribute to the distinct type I and II responses, for instance by inducing binding site specific conformations of the interacting protein complexes, as reported for the thyroid hormone receptor [34]. This is conceivable, since the 5′ extension characteristic of type II-associated PPREs has been shown to contribute to PPARγ-RXR binding by contacting a region adjacent to the zinc finger of the PPAR protein [26].

In contrast to genes showing type I or II responses, a clear consensus motif search in type III-associated peaks did not yield a defined consensus motif, but frequently imperfect PPREs or extended PPRE half-sites could be identified (Figure 5A; Dataset S10), suggesting that the role of PPARβ/δ may be to assist other factor(s) in transcriptional activation. However, at present we do not know whether these PPRE-like sequences are functionally relevant. It is, therefore, also possible that PPARβ/δ enrichment at some of the type III response genes is due to PPRE-unrelated mechanisms, resulting for instance from an interaction of PPARβ/δ with other DNA-binding transcription factors.

### Cell type-specific determinants

Several lines of evidence suggest that the regulation of PPARβ/δ target genes is not only determined by the genomic context, as shown in the present study, but also by cell type specific determinants. First, a comparison with published data shows that some genes characterized by a ligand-independent type I or type III response in WPMY-1 cells (Fig. 3A; Dataset S7) are inducible by PPARβ/δ agonists in other cell types, for example *IMPA2* in diploid human fibroblasts [22] or *HMOX1* in endothelial cells [35]. Second, the *PDPK1* and *ILK* genes, which are ligand-inducible in mouse keratinocytes [36], are unresponsive to ligand in WPMY-1 cells (Dataset S7). Although both genes respond to *PPARD* siRNA (Dataset S7), our ChIP-Seq analysis did not show an enrichment of PPARβ/δ binding at these loci (Dataset S1), suggesting that the siRNA effect in WPMY-1 cells is due to secondary events. Third, the *ANGPTL4* gene is strongly induced by PPARβ/δ in WPMY-1 cells (Fig. 3A) and other cell types [21]–[23], [33], [37], [38], but shows a type I response in HepG2 cells, even though other PPARβ/δ target genes (like *ADRP* or *CPT1A*) show a similar response in HepG2 and WPMY-1 cells (our unpublished observation). Taken together, these findings suggest that the cell type dependent availability or expression level of factors functionally and/or physically interacting with PPARβ/δ is an important co-determinant of the type of response of PPARβ/δ target genes.

### Correlation of the type of response with the biological function of target genes

Intriguingly, most validated genes showing a type II response (10/15; 67%) are directly associated with lipid metabolism, which is clearly different from the type I and III groups (5/24 and 0/0, respectively, corresponding to 21% and 0%). Likewise, Panther BP term classification identified a single significant hit across all three groups of genes, i.e. “lipid, fatty acid and steroid metabolism” for the type II response group with a *P* value of 0.0002 (Benjamini-Hochberg corrected) compared to *P* = 0.29 for type I response genes. These observations clearly point to a link between the biological function of PPARβ/δ target genes and their integration into a regulatory network.

### Conclusions

Our genomewide binding and expression studies with human myofibroblasts strongly suggest that PPRE-directed regulation by PPARβ/δ is not governed by a single mechanism. While repression in the absence of an agonistic ligand is commonly observed (with both type I and II responses), only the type II response involves an upregulation by ligand, which appears to be mediated to a major extent by release from transcriptional repression. Inspection of PPREs mediating type I and II responses suggests that their structure correlates with the differential response of the associated genes to ligands. On the other hand, genes showing a type III response are activated by PPARβ/δ in the absence of exogenous ligand, pointing to fundamentally different regulatory PPARβ/δ complexes. This scenario is reminiscent of the *Fabp4* gene in murine adipocytes which is constitutively activated by PPARγ in the absence of ligand, while under the same conditions the *Gyk* gene is ligand-dependent [9]. However, similar gene-specific responses have not been described for PPARβ/δ to date, although an agonist-independent association of PPARβ/δ with coactivators is structurally conceivable [39].

The existence of different modes of regulation suggests that PPARβ/δ is able to exert different biological functions, which is determined by the presence of ligands, its own expression level and the availability with specific coregulators. This hypothesis is supported by the results of our biological term classification, which showed that ligands affect primarily genes involved in lipid metabolism, suggesting that the biological function of PPARβ/δ target genes is indeed linked to the mode of their regulation. Taken together, these observations have important implications for elucidating the global PPARβ/δ signaling network and for understanding the function of ligand-based drugs in physiological and disease-related processes.

## Materials and Methods

### Cell culture and ligands

WPMY-1 cells [40] were obtained from the ATCC and maintained as described [21]. GW501516 and was from Axxora (Lörrach, Germany) and L165,041 from Calbiochem (Merck, Darmstadt, Germany).

### siRNA transfections

Cells were seeded at a density of 5×10^5^ cells per 6 cm dish in 4 ml DMEM with 10% FCS and cultured for 2 h. 1280 ng siRNA in 100 µl OptiMEM (Invitrogen) and 20 µl HiPerfect (Qiagen, Hilden, Germany) were mixed and incubated for 5–10 min at room temperature prior to transfection. The cells were replated 24 h post-transfection at a density of 5×10^5^ cells per 6 cm dish. Transfection was repeated 48 h after start of the experiment, and cells were passaged after another 24 h. Forty-eight hours following the last transfection, cells were stimulated and harvested after another 6 h.

### Quantitative RT-PCR

cDNA was synthesized from 0.1–1 µg of RNA using oligo(dT) primers and the Omniscript kit (Qiagen, Hilden, Germany). qPCR was performed in a Mx3000P Real-Time PCR system (Stratagene, La Jolla, CA) for 40 cycles at an annealing temperature of 60°C. PCR reactions were carried out using the Absolute QPCR SYBR Green Mix (Abgene, Hamburg, Germany) and a primer concentration of 0.2 µM following the manufacturer's instructions. *L27* was used as normalizer. Comparative expression analyses were statistically analyzed by Student's *t*-test (two-tailed, equal variance) and corrected for multiple hypothesis testing via the Bonferroni method. RT-qPCR primer sequences are included in Dataset S10.

### Microarrays

Microarray analyses were carried out as published [21]. Raw microarray data were normalized using the ‘loess’ method implemented within the marray package of R/Bioconductor [41]. Raw and normalized microarray data were deposited at EBI ArrayExpress (E-MEXP-2756). All data is MIAME compliant. Probes were considered regulated if they had an averaged log intensity > = 6 and a fold change > = 1.2 for GW501516 and > = 1.5 and siPPARD, respectively. Agilent microarray probes were aligned to both the reference genome (GRch37) and virtual cDNA created from Ensembl release 58 [42] and assigned to members of the Ensembl release 58 Homo sapiens gene set (see below). Genes with multiple probes passing the intensity threshold were assigned to the most strongly regulated probe for peak association.

### Assignment of microarray probes to genes

Agilent microarray probes were aligned to both the reference genome (GRch37) and in silico cDNA created from Ensembl release 58 (Hubbard et al. 2009) using bowtie 0.12.3, allowing 3 mismatches in the seed and 5 mismatches in total (Dataset S11). They were assigned to (possibly multiple) genes in the following order:

- perfect match in transcripts of a single gene,
- perfect match in transcripts of multiple genes, only one with matches within 2 kb of its transcripts 3′-ends,
- perfect genomic matches in different genes (no genes assigned),
- perfect genomic match to only one gene,
- perfect genomic match in one location covered by multiple genes,
- perfect genomic match outside of a gene with multiple matches to transcripts of different genes,
- perfect genomic match to a gene within 2 kb,
- perfect genomic match outside of a gene region (no gene assigned),
- mismatch in transcripts of a single gene,
- mismatch in transcripts of multiple genes, only one with matches within 2 kb of its transcripts 3′-ends,
- genomic mismatches in different genes (no genes assigned),
- mismatch to only one gene,
- mismatch in one location covered by multiple genes,
- mismatch outside of a gene with multiple matches to transcripts of different genes,
- mismatch to a gene within 2 kb,
- mismatch outside of a gene region (no gene assigned).

### ChIP-qPCR and ChIP Sequencing (ChIP-Seq)

ChIP-qPCR was performed and evaluated as described [21] using the following antibodies: IgG pool, I5006 (Sigma-Aldrich, Steinheim, Germany); α-PPARβ/δ, sc-7197; α-RXRα, sc-774; α-RNA polymerase II, sc-599; (Santa Cruz, Heidelberg, Germany); α-H3K4me3, pAb-003-050 (Diagenode, Liège, Belgium). Primer sequences are listed in Table S1. For ChIP-Seq, ChIP samples from WPMY-1 cells were sequenced on an Illumina IIx Genome Analyzer and analyzed with Bowtie [43] and MACS [44]. Sequencing data were deposited at EBI ArrayExpress (E-MTAB-371).

### Mapping of ChIP-Seq reads

Sequence reads (36 bp) were approximately deduplicated using a bloom filter (collision probability 10^−8^) and aligned to the human genome (GRch37) with bowtie 0.12.3 [43] allowing at most two mismatches (−n 2) with a mismatch quality sum of 70 (−e 70) and restricting to exactly one mapped location (−m 1 −k 1). Out of 33,078,626 PPARβ/δ total reads in three lanes, 20,777,020 unique reads could be aligned to distinct locations of the human genome (‘were mappable’); 1,553,813 failed to align. Out of 16,945,431 H3K4me3 reads in one lane, 10,183,043 were mappable and 935,169 failed to align. For RNA polymerase II (one lane) the numbers were 19,967,069, 10,248,554 and 2,178,974, respectively, and the three control IgG lanes yielded 62,436,651 total, 42,197,561 mappable and 3,394,035 failed reads.

### Peak finding

The aligned reads of multiple sequencing runs were combined as appropriate and passed to MACS [44] (--tsize = 36, --gsize = 2900000000 --mfold = 8) for peak finding, with the same IgG background used in both comparisons, PPARβ/δ and H3K4me3 (see Datasets S1 and S4 for results). To generate a high confidence PPARβ/δ peak set of 443 elements (Dataset S3), only peaks with a MACS-assigned FDR of less than 5% and fewer than 100 tags in the IgG track were selected. Furthermore, 17 peaks were removed from this set manually after visual inspection because of low signal-to-noise ratios. PPARβ/δ peaks without a H3K4me3 peak within 200 kbp were filtered, leading to the peak set titled “PPAR/H3K4”. RNA polymerase II occupancy was assessed for those H3K4me3 peaks having more than 50 Pol II tags in their region. Finally, we assigned the remaining 414 peaks to nearby regulated genes (see below: assignment of microarray probes), yielding a target gene set of 118 peaks corresponding to 112 genes (Dataset S8).

### Databases

The reference genome used throughout was the human genome assembly GRCh37 (http://www.ncbi.nlm.nih.gov/projects/genome/assembly/grc/human/index.shtml). All gene and transcript data, such as transcription start site positions, came from Ensembl revision 58 (http://may2010.archive.ensembl.org/). Functional annotation was retrieved from DAVID [32], genome wide association data from the supplemental data of [40].

### Comparison with single nucleotide polymorphism (SNP) data

Single nucleotide polymorphisms associated with phenotypes by genome wide association studies (GWAS) from reference [45] were extended by 200 kb on each side, and the percentage base pair overlap with the intervals of a query set (peaks) was measured. To evaluate the resulting scores, a Monte Carlo simulation with approximately *n = *10,000 trials was performed. Non-occurance within the trials was assigned a p-value of 3/N, and the result was corrected by the Benjamini-Hochberg procedure. The null model in the simulation retained the number of intervals, their sizes and their distance to the closest transcription start site in order to simulate random transcription factor binding sites. Enrichment was defined as the observed overlap divided by the mean overlap within the Monte Carlo simulation.

### Functional assignments

Gene sets were intersected with biological pathways as defined by Panther [31] (www.pantherdb.org) via the DAVID knowledge database [32] (http://david.abcc.ncifcrf.gov; release 6.7), using Ensembl gene ids as the main identifier. P-value was assessed by a corrected hyper geometric test (DAVID's EASE score) and correction for multiple hypothesis testing was done via the Benjamini-Hochberg procedure.

### Motif search

*De novo* motif search was performed using MEME (version 4.3.0) [25]. MEME parameters were “–dna –mod zoops –minw 10 –maxw25 –maxsize 100000 –revcomp –p 7” for Fig. 2C (FDR = 0 motif) and “–dna –mod zoops –minw 17 –maxw17 –maxsize 100000 –revcomp –p 7” for subsequent motif searches.

## Supporting Information

Figure S1 **Detection of PPARβ/δ, H3K4me3 and RNA polymerase II enrichment peaks at the** ***SLC25A20*** **locus by ChIP-Seq.**
(TIFF)

Figure S2 **Detection of PPARβ/δ, H3K4me3 and RNA polymerase II enrichment peaks at the** ***CDKN2C*** **locus by ChIP-Seq.**
(TIFF)

Figure S3 ***PPARD*** **siRNA-mediated inhibition of ligand-induced transcriptional activation of PPARβ/δ.** WPMY-1 cells were transfected with a PPRE-luciferase construct in the presence of control or *PPARD* siRNA and treated with GW501516 for 24 hrs. The knockdown effect was abolished by cotransfection of a *PPARD* expression vector.
(TIFF)

Figure S4 **Efficiency and specificity of the siRNA-mediated knockdown of** ***PPARA*** **,** ***PPARG*** **and** ***PPARD*** **.** WPMY1-1 cells were transfected with the indicated siRNA pools or control siRNA (si-con) and relative expression levels of *PPARA, PPARG* and *PPARD* were measured by RT-qPCR.
(TIFF)

Table S1 **Primers used for RT-qPCR analyses.**
(PDF)

Dataset S1 **ChIP-Seq data set of all PPARβ/δ enrichment peaks (complete list;** ***n =*** **4,542).**
(XLS)

Dataset S2 **Comparison of PPARβ/δ enrichment peaks with SNP data.**
(XLS)

Dataset S3 **High confidence PPARβ/δ peak set (** ***n =*** **443), consisting of peaks with a MACS-assigned FDR of less than 5% and fewer than 100 tags in the IgG track.**
(XLS)

Dataset S4 **ChIP-Seq data set of histone H3 lysine-4 trimethylation (H3K4me3) enrichment peaks (complete list;** ***n =*** **24,843).**
(XLS)

Dataset S5 **ChIP-Seq data set of RNA polymerase II enrichment in genes (complete list).**
(XLS)

Dataset S6 **Microarray analysis of WPMY-1 cells treated with** ***PPARD*** **or control or siRNA (complete list).**
(XLS)

Dataset S7 **Microarray analysis of WPMY-1 cells exposed to GW501516 or solvent (complete list).**
(XLS)

Dataset S8 **“Target gene set” defined by correlating ChIP-Seq and microarray data (** ***n =*** **112).**
(XLS)

Dataset S9 **Panther Biological Pathway term analysis of the target gene set.**
(XLS)

Dataset S10 **Data set of validated target genes (** ***n =*** **53), including primers used for RT-qPCR analyses.**
(XLS)

Dataset S11 **Alignment of Agilent microarray probes to both the reference genome (GRch37) and in silico cDNA created from Ensembl release 58.**
(XLS)
