# Supplementary figures and images for: Genomewide Analyses Define Different Modes of Transcriptional Regulation by Peroxisome Proliferator-Activated Receptor-β/δ (PPARβ/δ)
Source: PLoS One. 2011 Jan 19;6(1):e16344. doi: 10.1371/journal.pone.0016344 (PMC3023804; doi:10.1371/journal.pone.0016344)

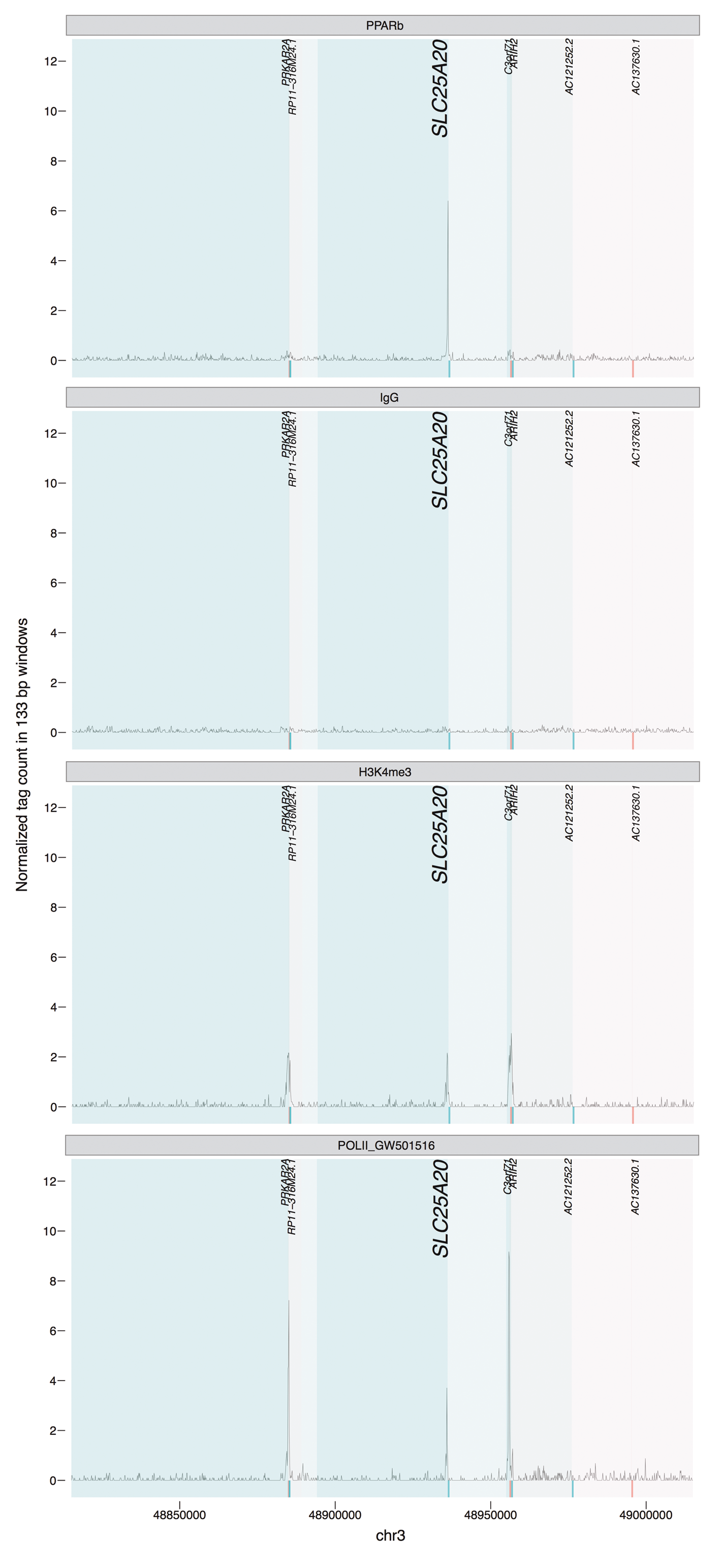

Supplement: Figure S1 — Detection of PPARβ/δ, H3K4me3 and RNA polymerase II enrichment peaks at the SLC25A20 locus by ChIP-Seq. (TIFF) [file pone.0016344.s001.tif]

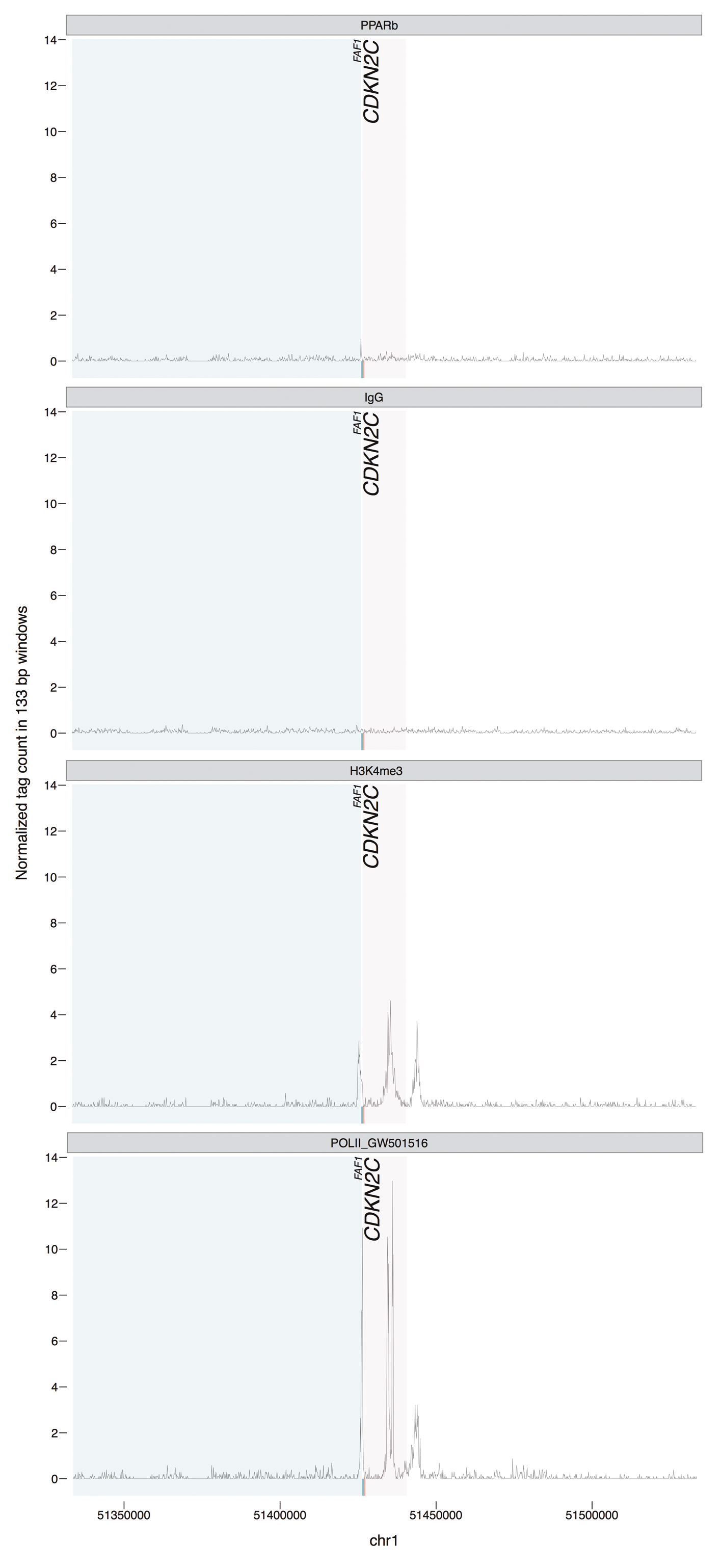

Supplement: Figure S2 — Detection of PPARβ/δ, H3K4me3 and RNA polymerase II enrichment peaks at the CDKN2C locus by ChIP-Seq. (TIFF) [file pone.0016344.s002.tif]

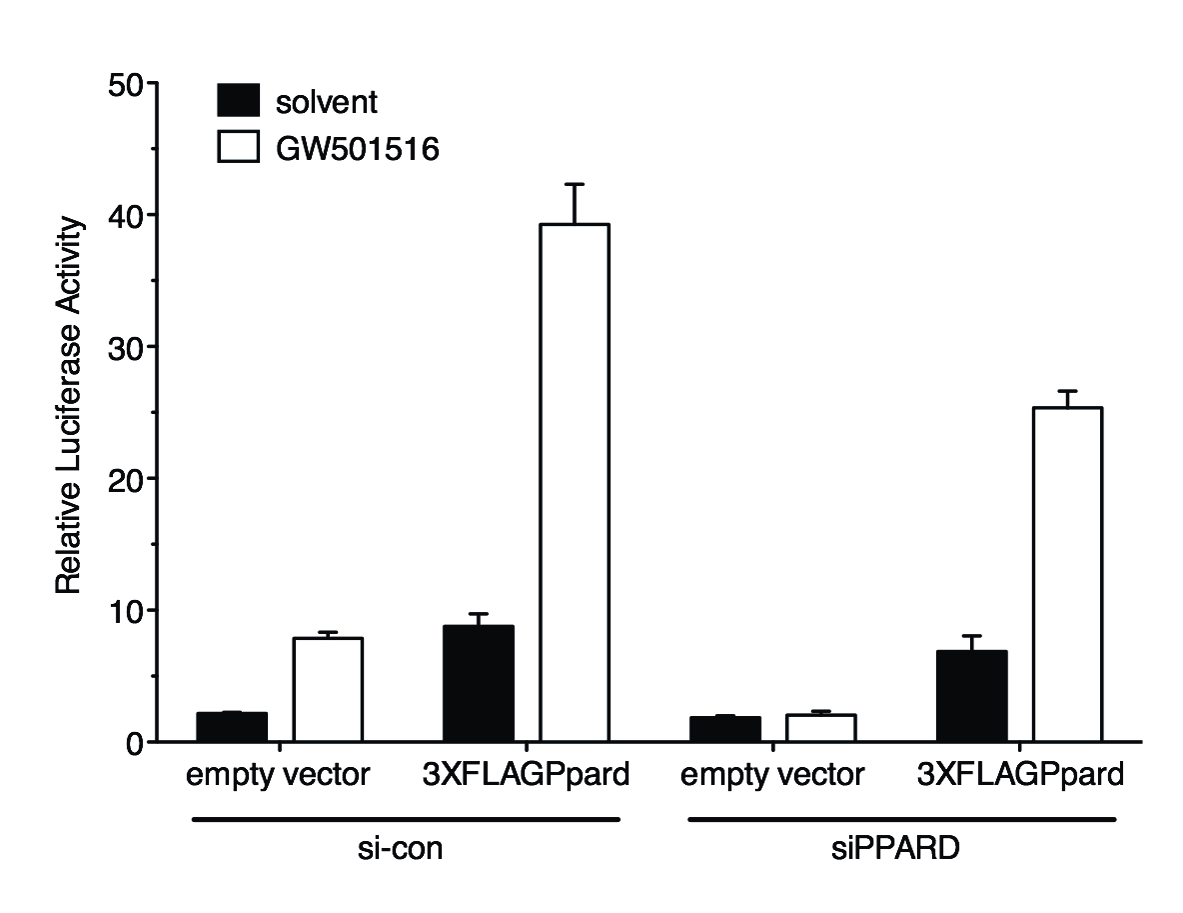

Supplement: Figure S3 — PPARD siRNA-mediated inhibition of ligand-induced transcriptional activation of PPARβ/δ. WPMY-1 cells were transfected with a PPRE-luciferase construct in the presence of control or PPARD siRNA and treated with GW501516 for 24 hrs. The knockdown effect was abolished by cotransfection of a PPARD expression vector. (TIFF) [file pone.0016344.s003.tif]

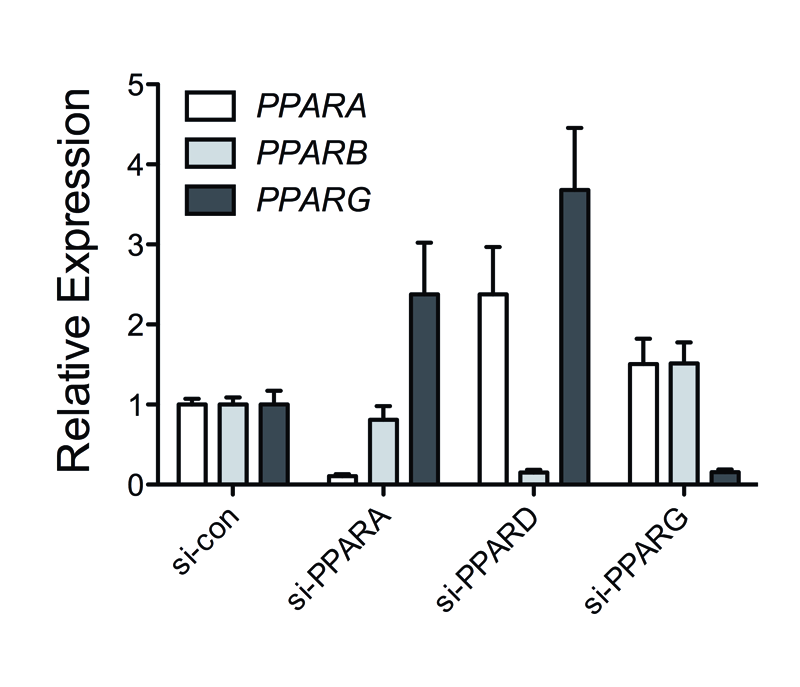

Supplement: Figure S4 — Efficiency and specificity of the siRNA-mediated knockdown of PPARA , PPARG and PPARD . WPMY1-1 cells were transfected with the indicated siRNA pools or control siRNA (si-con) and relative expression levels of PPARA, PPARG and PPARD were measured by RT-qPCR. (TIFF) [file pone.0016344.s004.tif]
