# Supplementary material for: Genomewide Analyses Define Different Modes of Transcriptional Regulation by Peroxisome Proliferator-Activated Receptor-β/δ (PPARβ/δ)
Source: PLoS One. 2011 Jan 19;6(1):e16344. doi: 10.1371/journal.pone.0016344 (PMC3023804; doi:10.1371/journal.pone.0016344)
Supplement: Table S1 — Primers used for RT-qPCR analyses. (PDF) [file pone.0016344.s005.pdf]

Table S1.

Primers used for RT-qPCR analyses.

Primer	Sequence (5’ → 3’)
ABCA1fw	GTAGGTGAAAGGTAACTAGCAGACG
ABCA1rv	GCGAAGTGCAGACTTGGAG
AHNAKfw	GCGAGGACGAAACAGAAGAC
AHNAKrv	AATTCAGCGCCACTGGATAC
IMPA2fw	CGGCCGTATTTACCAGCTTA
IMPA2rv	GTGAAAAAGGACTCGCCAAC
CYB5D2fw	GGCCTCTACCAACTCACGAC
CYB5D2rv	TCGTCCTCTGGGAAATCAAG
BIRC3fw	TGAGAACTCGAAGGTCTTGTTTC
BIRC3rv	TTGCTCTTTATGCCGATGG
BAI2fw	AAAAGCAACCCCAAGGAAAC
BAI2rv	AAGCCTGACCTCTGCTCTTG
PPARG_1fw	AGCTCCCACTGGGGAAATAC
PPARG_1rv	GGATGTGACCTTTCCCTTAGATT
PPARG_2fw	CCTGGGCAGAGAACCAAGTA
PPARG_2rv	CTTCAGAGAAACCCCACCTTC
GPR180fw	ACATCTCCCAGCTCAGCAGT
GPR180rv	TGCGCTTTTCAGACCATACTC
CDKN2Cfw	GTCGCACATTTTGCCTGTC
CDKN2Crv	CTTGACAGGAGGGTGGTCA
HSDL2fw	TAGGTGAAAGGTCGCAGGAG
HSDL2rv	GAATTGAGCGTCAGGAACAAG
MLYCDfw	TTAGGCGGTCAGGTGTTAGG
MLYCDrv	CCAAGTGTGGCTAAAGGTTACTG
SLC25A20fw	TGATGGGTTTTGGCTGGT
SLC25A20rv	AGAACTGACAGACGGAGTGACA

ADRP_1fw	GAAAAATCAACTGGCGGAAA
ADRP_1rv	CCCCACATGCCCAATAATAC
ADRP_2fw	CCATCCCTGCTCTAATGACC
ADRP_2rv	AGGGCGAGAGTCTTCTGATG
ADRP_3fw	TCCCATGCACAAAGTGAAAG
ADRP_3rv	GCATACCAGCATCTTGAGCA
AC009226.3fw	CCAACATCACATGCTTCCTG
AC009226.3rv	GAGGTGGTGGAGTGGTGACT
ACAA2fw	GCAATCACCCAGATTTCAAGA
ACAA2rv	AGCCCCAGGAAGTAGGTGA
ACADVLfw	AAAGTAGGGGAAAGGGCAAG
ACADVLrv	AGAGACGGTTAGCAAGTTCAGG
AP2A1fw	TTCCATCTCCCAGGATTCAG
AP2A1rv	ACAAAACTCCACCCCATCAG
AV704385fw	CAGCCTCAGCATTCATAAGTTG
AV704385rv	CAAAGGTGACGGGGTATGAC
CPT1Afw	CTCCGGAAGGTCTCTGTGG
CPT1Arv	AAAGTAGGGGAAAGGTCAGCA
CRLS1fw	GGCTTCCTGCCACCTGTAT
CRLS1rv	ACTCAGACACTGGGCTACCG
DIAPH1fw	AAAGTCCATAGGCTGCCAGA
DIAPH1rv	AAGCAAATGAGGGTGGAATG
E4F1fw	GTCGTAAATCCGCCATCTTC
E4F1rv	CTGGGCTTCTGCCGTATG
ELP3fw	CCGGGAAGAGCTTTACGATAC
ELP3rv	AATGCAGCCACAAACTCAGAC
RAD52fw	AGCGTCTCTGGGAAGAAGGT
RAD52rv	TCTTTTCCCCTCCGACTTG
RP11-245M24.1fw	TTTGCCAAAGCCAACATTC
RP11-245M24.1rv	TTGACAGGAAAAGCTTTCAGC

NCOA5fw	AAGACCGCGCGTGTTTAC
NCOA5rv	AGCCTAGGACGCTGTTGCTA
KLF10fw	CGAGGCATGTGAACAAAGC
KLF10rv	GGCAATTCCCAGTTCACG
LEO1fw	CTCTCTTTACGGCACGGAAC
LEO1rv	GCGAGCGAAAACTAGGTCAC
NAPfw	AAGCAACCCTGTTCCAAATG
NAPrv	CAGAGCCAATAGGGCAAAAG
NUDT9fw	GACTGGGAAGTCTGGGAAGTC
NUDT9rv	GGCCACGAACCTCGTAACT
PI4KBfw	CGGGGAGTAAAGATGAAGCA
PI4KBrv	GAAATCTGCCGCATGTCAC
PSMB6fw	GTGCTCACTGTCGCAAAAGA
PSMB6rv	ACTGTCGTAAAGCGCTCTGTC
TNPO3fw	GCCACACACCAGTGTACCTAAG
TNPO3rv	GGAAGGAGGCCGTTTGTAG
ZMAT2fw	TGGAGGGCTATCTTGCCTTA
ZMAT2rv	TGAAAAACGCCGTAAAAAGC
ZNHIT6fw	TCGAATCGCCGTAAAGCTAC
ZNHIT6rv	TCACGCAGTTACGCTTGTTC
PDK4-12000fw	GCAGAGTCAACAAGGGGAAG
PDK4-12000rv	ACTAGATGCCTGGGAGCTGA
control fw (PDK4 “RE2”)	TTCTATGTGCTGTGGGCTGA
control rv (PDK4 “RE2”)	TTTGGCAACCTCAGTTCACA
ANGPTL4+3500fw	CCTTACTGGATGGGAGGAAAG
ANGPTL4+3500rv	CCCAGAGTGACCAGGAAGAC
